# Predictors of Acute Diarrhoea among Hospitalized Children in Gaza Governorates: A Case-Control Study

**Published:** 2015-03

**Authors:** Samer Khader Alnawajha, Ghadeer Abdo Bakry, Yousef Ibrahim Aljeesh

**Affiliations:** ^1^University College of Applied Sciences, Ibn Sina st. Rafah, Gaza, Palestine; ^2^Palestine College of Nursing, Ibn Sina st. Rafah, Gaza, Palestine; ^3^Islamic University of Gaza, Remal, Gaza, Palestine

**Keywords:** Diarrhoea, Hospitalized children, Predictors, Gaza Strip

## Abstract

This study aims to determine the predictors of acute diarrhoea among hospitalized children in the Gaza Governorates. The case-control design included 140 children (70 cases and 70 controls) in a stratified cluster sample from Naser Medical Complex and Alnasser Pediatric Hospital. An interview questionnaire was used, and face and content validations were performed. Multiple logistic regression was used for the multivariate analysis of risk factors of diarrhoea in children aged less than five years. Results showed a significant association between diarrhoea and family income, residence, complementary feeding, and age of weaning (p<0.05). Children living in villages had lower odds of having diarrhoea by 53.2% than children living in cities. Children of families with incomes between US$ 485 and 620 had lower odds of having diarrhoea by 80.8% than children of families with incomes less than US$ 485. Moreover, children who did not receive complementary feeding had lower odds of having diarrhoea by 59.0%. We found that, for one month increase in weaning age, the odds of diarrhoea decreased by 1.06 times (adjusted OR=1.05, 95% CI 1.0180-1.100). The study concludes that urban residence, lower family income, complementary feeding, and lower age of weaning are risk factors of diarrhoea among children aged less than five years in the Gaza Strip. The results of the study suggest that children of low-income families and those who were not naturally breastfed may warrant more attention for prevention and/or treatment of diarrhoea.

## INTRODUCTION

Globally, diarrheoal disease is the second leading cause of death in children below five years of age and is responsible for killing around 760,000 children every year (1). Malnourished children or children who have impaired immunity are most at risk of life-threatening diarrhoea, which usually results from an intestinal tract infection that can be caused by various bacterial, viral and parasitic organisms. Infection can spread through contaminated food or drinking-water or from person-to-person as a result of poor hygiene (2). Acute diarrhoea is an abnormal frequent discharge of fluid stool, or semi-solid matter from the bowel lasting less than 14 days (3). This problem is common in the Gaza Strip as found in a health survey of the West Bank and Gaza Strip. In total, 14% of children aged less than five years had an episode of diarrhoea in the two weeks preceding the survey (4). The percentage of dehydration among children suffering from diarrhoea in the Gaza Strip is 6% (4). Moreover, the highest percentage of diarrhoeal disease was found among infants aged less than six months (23%) (4). The incidence of diarrhoea in children aged less than three years increased during the fourth quarter of 2011, during which the incidence was 20% (5). Many children aged less than five years were admitted to governmental hospitals because of diarrhoea (4). These diarrhoeal episodes among children require research to determine the risk factors that led to the disease. Available information on the risk factors of diarrhoea among children in Gaza is limited. In this study, we aimed to explore the risk factors associated with diarrhoea among children aged less than five years. The results highlight modifiable and other risk factors, which may be useful to public health policy-makers or programmes that aim to prevent diarrhoea among young children in Gaza.

## MATERIALS AND METHODS

### Setting

The study was carried out in the Gaza Strip at the Naser Medical Complex, Alnasser Pediatric Hospital, Khanyounis Primary Health Care Center, and Sheikh Radwan Primary Health Care Center, from June to November 2012.

### Study population

The target population consisted of two groups. The first group included cases in children who were hospitalized because of acute diarrhoea. Diarrhoea was characterized by three or more loose bowel movements a day or passage of blood in the stool in the preceding two weeks as reported by the caretaker. The case-children were aged less than five years and stayed in the Pediatric Ward at the Naser Medical Complex and Alnasser Pediatric Hospital in June and July 2012. Children in the second group were of the same age and served as controls that consulted the Khanoyounis and Sheikh Radwan primary healthcare clinics for scheduled vaccinations. The medical facilities that served the case and the control groups were close to each other and served the same population within the same area. The controls had not experienced diarrhoea since January 2012. Controls with previous hospitalization for diarrhoea were excluded from the study.

### Sample-size and sampling method

The study included a stratified cluster sample of all those who came for treatment during the specified period. The Gaza Strip was divided into two areas: south and north. The Naser Medical Complex represented the south area, and the Alnasser Pediatric Hospital represented the north area. The sample was calculated using Epi Info software (version 3.0.43). Based on literature review and consultation with experts in the field of study, the highest risk factor of diarrhoea was known to be malnutrition, which was 30% among the ill group and 10% among the healthy group (6). Based on the calculation using Epi Info, the sample-size was 140 (70 cases and 70 controls) at α=0.05 and power=0.8. A combination of measures (weight, height, and BMI) was considered. Controls were matched with cases, based on gender and age (1 month to 1 year, 1.1 years to 2 years, and 2.1 years to 4.6 years).

### Procedure for data collection

The questionnaire was submitted to the panel of experts for suggestions and judgement on the adequacy of the instrument. Construct validation was also performed.

The subjects were recruited based on the selection criteria. Information on the following was recorded on a close-ended proforma used in an interview with mothers. Detailed history was recorded, including data on age, sex, education, and occupation of parents; health and nutritional status of the child, such as weight and height; family information, such as type of family, status of parents, and average family income per month; household characteristics, such as main source of water, main source of drinking-water, household wealth index quintile, place of residence, type of access to toilet facilities, and method of disposal of the child's faeces; and child-feeding practice, such as duration of breastfeeding, age of weaning, and number of meals per day. Trained doctors, accompanied by nurses, carried out the questionnaire during the study.

### Measurements

All anthropometric measurements, such as weight, height, body mass index (BMI), weight-for-age z-score, and height-for-age z-score were recorded using standardized procedures. BMI was calculated based on the WHO standards. Subjects were considered overweight when BMI was ≥25.0 kg/m^2^, underweight when BMI was <18.5, obese when BMI was ≥30.0, and normal when BMI was 18.50 to 24.9. The z-score is defined as the standard deviation (SD) from the mean when the distribution is normal (6). Weight-for-age z-score is an indicator of nutritional status relating to weight and age of a child expressed in SD from the median reference value of the National Center for Health Statistics-Centers for Disease Control and Prevention (NCHS-CDC), with classification of severely underweight (<− 3 SD), moderately underweight (−3 to −2.01), overweight (≥2.0 SD), and normal (>−2.0) (7). Height-for-age z-score is an indicator of nutritional status related to height and age of a child expressed in SD from the median reference value of the (NCHS-CDC) with classification into severely stunted (<−3 SD), normal (≥−2 SD), and moderately wasted (−3 to −2.01) (7).

The current status and diagnosis of diarrhoea was obtained from the child's medical record, history from the caretaker (mother), and assessment by specialist doctors of paediatrics in the study hospitals. Child-feeding practices were classified as: exclusive breastfeeding, complementary feeding, and replacement feeding. Exclusive breastfeeding referred to feeding breastmilk only or breastmilk with vitamins, mineral supplements, and medicines. Complementary feeding referred to feeding breastmilk and solid or semi-solid food. Replacement feeding (weaned) referred to feeding foods other than breastmilk.

### Data analysis

Data were analyzed using the PASW software (version 18). Mean, SD, and percentages were used for the descriptive analysis. Univariate logistic regression analysis was conducted by comparing the outcome variable (diarrhoea), with each independent variable of interest, such as age, sex, residence, parents’ age and education, type of children (orphan and vulnerable), risk factors of household environment, and others, using odds ratio (OR) and their 95% confidence interval (CI). The likelihood ratio test was used in estimating OR and 95% CI for all associations of interest. Multivariate logistic regression analysis was performed to adjust for simultaneous effects of multiple factors and to control the effects of confounding factors on the outcome variable. To form the best fitting and the most parsimonious but biologically-sound model, variables with p<0.25 (8) by univariate analysis, those that were clinically important to all children aged below five years, and those that had statistically significant association with diarrhoea were selected as predictors, along with all the variables of known clinical importance and included in the multivariate model. Wald statistic for each variable was used in assessing the importance of each variable included in the model. The parameters of the logistic regression model were estimated by the maximum likelihood method.

### Ethical approval

The study followed all the ethical considerations required to conduct a medical research. Ethical approval was obtained from the School of Public Health of the Al-Quds University. The Helsinki Committee in Gaza-Palestine approved the study protocol and instrument. An approval letter was obtained from the Ministry of Health in the Gaza Strip to visit the hospitals and the primary healthcare centres. Informed consent was obtained from the respondents. The details of the study methodology were explained to the subjects. The subjects were recruited based on the selection criteria.

## RESULTS

A sample of 140 subjects (70 cases and 70 controls) was enrolled. Seventy children (50%) (35 cases and 35 controls) were from Gaza, and 70 (50.0%) (35 cases and 35 controls) were from Khanyounis. Fifty percent of cases were male, and 50% of the controls were female. Their ages ranged between 1 month and 4.6 years, mean age was 1.38, and SD was ±1.39. Twenty-six (50.0%) from the age-group between 1.1 years and 2 years were cases, and 26 (50.0%) from this group were controls whereas 34 (50.0%) from the age-group of 1 month to 1 year were cases, and 34 (50.0%) were controls. Ten (50.0%) from the age-group of 2.2 years to 4.6 years were cases, and 10 (50.0%) were controls. Among those who were living in cities, 31 (37.8%) were cases, and 51 (62.2%) were controls. Among those who were living in villages, 23 (56.1%) were cases, and 18 (43.9%) were controls. Among those who were living in camps, 16 (94.1%) were cases, and one (5.9%) was control (Table 1).

The adjusted ORs and their 95% CIs were computed using the estimates of the parameters of the final model. The probability (p) level of less than 0.05 was considered significant. Model fitness was verified by Hosmer-Lemeshow's goodness-of-fit test, classification table, and area under receiving operating characteristics curve. The null hypothesis for Hosmer-Lemeshow's goodness-of-fit test of the model was fit, and the p value was 0.926, which is not significant. Therefore, the model is fit. Moreover, the classification table by SPSS showed that 72.1% of cases were predicted accurately whether they had diarrhoea or not (70% or above is considered a good model) (8). None of the interactions was significant, and collinearity between the two variables was verified by linear regression. The variance inflation factor for each independent variable was 1.033, which is less than the considered acceptable value of 10 (8).

The results of univariate analysis showed that the hospitalized children aged less than 5 years, who were living in camps (OR=0.03, 95% CI 0.005-0.301), had an average family income of US$ 485 to 620 (OR=0.19, 95% CI 0.070-0.554), had no income (OR=0.21, 95% CI 0.083-0.564), had an unimproved toilet facility (OR=0.31, 95% CI 0.116-0.865), were exclusively breastfed (OR=2.14, 95% CI 1.088-4.246) (Table 2), had a weight-for-age z-score >2.0 (OR=5.28, 95% CI 1.074-25.956), and height-for-age z-score >2.0 (OR=5.00, 95% CI 1.340-18.655) were significantly susceptible to diarrhoea (Table 2). Eating 8 to 12 meals per day (OR=0.11, 95% CI 0.013-0.977), receiving complementary feeding (OR=0.49, 95% CI 0.252-0.976), and increase in weaning age (OR=1.03, 95% CI 1.008-18.069) are statistically significant protective factors of diarrhoea among hospitalized children aged less than five years (Table 2).

**Table 1.** Sociodemographic characteristics of cases and controls (N=140)

Characteristics	Cases N (%)	Controls N (%)	Total N (%)	Characteristics	Cases N (%)	Controls N (%)	Total N (%)
Gender				OVC^1^ status			
Male	37 (50.0)	37 (50.0)	74 (52.9)	Orphaned	1 (1.4)	2 (2.9)	3 (2.1)
Female	33 (50.0)	33 (50.0)	66 (47.1)	Vulnerable	4 (5.7)	3 (4.3)	7 (5.0)
Age				Not OVC	65 (92.9)	65 (92.9)	130 (92.9)
1 month–1 year	34 (50.0)	34 (50.0)	68 (48.6)	Source of drinking-water			
(1.1-2) years	26 (50.0)	26 (50.0)	52 (37.1)	Filter	16 (22.9)	25 (35.7)	41 (29.3)
(2.1-4.6) years	10 (50.0)	10 (50.0)	20 (14.3)	Mineral water	47 (67.1)	40 (57.1)	87 (62.1)
Governorate				Municipality water (not filtered)	7 (10.0)	5 (7.1)	12 (8.6)
Khanyounis	35 (50.0)	35 (50.0)	70 (50.0)	Family type			
Gaza	35 (50.0)	35 (50.0)	70 (50.0)	Nuclear	46 (65.7)	50 (71.4)	96 (68.6)
Residence				Extended	24 (34.3)	20 (28.6)	44 (31.4)
City	31 (37.8)	51 (62.2)	82 (58.6)				
Village	23 (56.1)	18 (43.9)	41 (29.3)				
Camp	16 (94.1)	1 (5.9)	17 (12.1)				
Income per month (US$)				Mother's age			
Less than 485	9 (12.9)	25 (35.7)	34 (24.3)	17-25 years	34 (48.6)	33 (47.1)	67 (47.9)
485-620	22 (31.4)	12 (17.1)	34 (24.3)	26-34 years	29 (41.4)	23 (32.9)	52 (37.1)
More than 620	9 (12.9)	15 (21.4)	24 (17.1)	35-43 years	7 (10.0)	14 (20.0)	21 (15.0)
No income	30 (42.9)	18 (15.7)	48 (34.3)	Father's age			
Mother's education				20-30 years	33 (47.1)	37 (52.9)	70 (50.0)
School	48 (68.6)	52 (74.3)	100 (71.4)	31-40 years	34 (48.6)	22 (31.4)	56 (40.0)
University	22 (31.4)	18 (25.7)	40 (28.6)	41-59 years	3 (4.3)	11 (15.7)	14 (10.0)
Father's education				Mother's work			
School	52 (74.3)	47 (67.1)	99 (70.7)	Employed	6 (8.6)	4 (5.7)	10 (7.1)
University	18 (25.7)	23 (32.9)	41 (29.3)	Unemployed	64 (91.4)	66 (94.3)	130 (92.9)

^1^OVC=Orphans and vulnerable children

No significant association was found between father's educational level and diarrhoea (chi-square value=7.370 and p=0.195). The father's age and diarrhoea were significantly associated (chi-square value=7.371 and p=0.025, Table 2).

Variables with p<0.25 (8) were selected as predictors, along with all the variables of known clinical importance and included in the multivariate model, These were: residence, toilet facility, exclusive breastfeeding, complementary feeding, weight-for-age z-score, height-for-age z-score, previous hospitalization, body mass index, father's age, family income, source of drinking-water, faeces disposal method, number of meals per day, and weaning age.

The final multivariate logistic regression model revealed that the children who are living in camps had lower odds of having diarrhoea by 99.9% than children who were living in cities (adjusted OR=0.03, 95% CI 0.005-0.326). Moreover, children of families with incomes of US$ 485 to 620 had lower odds of having diarrhoea by 80.8% than children of families with incomes less than US$ 485 (adjusted OR=0.19, 95% CI 0.057-0.644). Children of families with no income had lower odds of having diarrhoea by 68.9% than children of families with incomes less than US$ 485 (adjusted OR=0.31, 95% CI 0.102-0.950).

**Table 2.** Univariate logistic analysis of the factors associated with diarrhoea among children aged less than five years

Variable	Diarrhoea		OR	95% CI	p value
	Cases No (%)	Control No (%)			
Residence					
City^®^	31 (44.3)	51 (72.9)	1	-	-
Village	23 (32.9)	18 (25.7)	0.47	0.222-1.019	0.056
Camp	16 (22.9)	1 (1.4)	0.03	0.005-0.301	0.002**
Family income (US$)					
Less than 485^®^	9 (12.9)	25 (37.7)	1	-	-
(485-620)	22 (31.4)	12 (17.1)	0.19	0.070-0.554	0.002**
More than 620	9 (12.9)	15 (21.4)	0.60	0.195-1.846	0. 373
No income	30 (42.9)	18 (25.7)	0.21	0.083-0.564	0.002**
Type of toilet facility					
Improved^®^	54 (77.1)	64 (91.4)	1	-	-
Unimproved	16 (22.9)	6 (8.6)	0.31	0.116-0.865	0.025
Number of meals per day					
1-3 meals^®^	26 (37.1)	39 (55.7)	1	-	-
4-7 meals	38 (54.3)	30 (42.9)	0.526	0.264-1.049	0.068
8-12 meals	6 (8.6)	1 (1.4)	0.11	0.013-0.977	0.048
Exclusive breastfeeding (in the first 6 months)					
No^®^	46 (65.7)	33 (47.1)	1	-	-
Yes	24 (34.3)	37 (52.9)	2.14	1.088-4.246	0.028*
Complementary feeding					
No^®^	25 (35.7)	37 (52.9)	-	-	-
Yes	45 (64.3)	33 (47.1)	-0.49	0.252-0.976	0.042*
Weight-for-age z-score					
Severely underweight (<−3)^®^	8 (11.6)	2 (2.9)	1	-	-
Moderately underweight (−3 to −2.01)	11 (15.9)	2 (2.9)	0.72	0.0846.314	0.773
Normal (>−2.0)	50 (72.5)	66 (94.3)	5.28	1.074-25.956	0.040*
Height-for-age z-score					
Severely stunted (<3)^®^	12 (17.1)	3 (4.3)	1	-	-
Moderately stunted (−3 to −2.01)	6 (8.6)	2 (2.9)	1.33	0.173-10.254	0.782
Normal (>−2.0)	52 (74.3)	65 (92.9)	5.00	1.340-18.655	0.017*

OR=Odds ratio

CI=Confidence interval

^®^Reference group

*Significant at 0.05 level

**Significant at 0.01 level

The results revealed that the children who did not receive complementary feeding had lower odds of having diarrhoea by 59.0% than children who received such feeding (adjusted OR=0.41, 95% CI 0.171-0.983). We found that, for one month increase in weaning age, the odds of diarrhoea decreased by 1.06 times (adjusted OR=1.05, 95% CI 1.0180-1.100).

## DISCUSSION

The prediction model of having diarrhoea among children aged less than five years is:

Logit (P)=In{P/1-P}=−0.432–{3.260*living in camp}–{1.651*income (US$ 485 to 620)}–{0.702*did not receive complementary feeding}−{0.037*increased age of weaning}.

**Table 3.** Multivariate logistic analysis of the factors associated with diarrhoea among children aged less than five years

Variable	Adjusted OR^2^	(95% CI)^3^	p value
Residence			
City^®^	1	-	-
Village	0.46	(0.189-1.156)	1.000
Camp	0.03	(0.005-0.326)	0.003**
Family income (US$)			
Less than 485^®^	1	-	-
(485-620)	0.19	(0.057-0.644)	0.008**
More than 620	0.86	(0.226-3.311)	0.833
No income	0.31	(0.102-0.950)	0.040^®^
Complementary feeding			
Yes^®^	1	-	-
No	0.41	(0.171-0.983)	0.046*
Weaning age^1^	1.05	(1.018-1.100)	0.005**

^1^Numerical variable

^2^Multiple logistic regression

^®^Reference group

^3^CI=Confidence interval

*Significant at 0.05 level

**Significant at 0.01 level

The model reasonably fits well. Model assumptions are met. There are no interaction and multicollinearity problems

These results are not consistent with that of Girma *et al*. (9), which showed that absence of refuse disposal facility and presence of faeces around the pit-hole were independently associated with diarrhoeal morbidity among under-five children in the multivariate logistic regression model. The model was used for evaluating associations between factors and dichotomous variables that were designed to measure environmental risk factors of morbidity among under-five children during the two weeks prior to the survey (9).

Wilund and Panza included wealth index quintile, nationality of household head, educational level of household head, main source of drinking-water, number of under-five children in the household, caretaker's age, and child's gender and age in their model (10). Based on this, the differences in regions made differences in the factors associated with diarrhoea for under-five children because of different sources of drinking-water and different educational levels of parents, and others. Poverty has been related to unemployment, crowdedness, and poor interactions, which all reflect a family's health status. In addition, sufficient family income has been reported as a main factor that leads to better health outcomes (11). Our findings are consistent with those of several studies which had found significant association between household economic status and diarrhoea in children (12–14). Generally, children living in poor households have higher rates of infection with diarrhoea than their wealthier counterparts, probably because of inadequate access to sanitary facilities, unsanitary environments in the home, and poor hygiene of the child. In this study, the children with lower income had lower odds of having diarrhoea than those with higher family income. This may be attributed to a higher likelihood of children with higher family income in Gaza Governorates to eat chocolate, sweets and received complementary feeding as an extra food. This could result in bowel changes which lead to diarrhoea.

Our results are consistent with the findings of Mahalanabis *et al*. who reported that, in their logistic regression model, the effect of maternal education remained high after adjustment for several confounders (15). Based on the concept that socioeconomic variables operate through a set of proximate variables, maternal education, independent of economic power, favourably influences child survival through its impact on disease from acute diarrhoea.

Most families in this sample (29.3% in villages and 12.1% in camps) lived in residences with one to two room(s) and 15.7% had poor or moderate household hygienic conditions because of unimproved toilet facilities and lack of safe sewage disposal. Lack of access to preventive health information and limited rights to access services can be risk factors of morbidity in the affected population (6). Thus, the outcome of this study should not be considered unexpected. In our study, no significant association was observed between source of drinking-water and diarrhoeal morbidity. This finding is likely because of that heterogeneity among the sample households with respect to the sources of drinking-water used. Contrary to this finding, source of drinking-water is an important environmental determinant of diarrhoeal morbidity as previously reported (16–18).

### Limitations

We did not use conditional logistic regression in the statistical analysis that may not have accounted for bias due to confounders. The survey did not include questions about handwashing which has been shown to affect childhood diarrhoea (2).

### Conclusions

This study found that living in camps, having household income of US$ 485 to 620 not receiving complementary feeding and having an increased age of weaning were the main factors associated with diarrhoea among hospitalized children aged less than five years in Gaza Governorates.

### Recommendations

Improving health education about diarrhoeal disease in remote areas of Gaza by encouraging health policy and decision-makers to visit families could help reduce risk in rural areas. In addition, special health education programmes for mothers and/or caretakers that promote proper feeding practices, attention to nutritional status according to WHO laws and policies, and encouraging breastfeeding may also help reduce risk of diarrhoea among young children. Since low income was associated with childhood diarrhoea, extension of social and financial support to families who have no family income and those who have low income may also be a helpful measure.

## ACKNOWLEDGEMENTS

The authors would like to thank Associate Professor Yousef Aljeesh for reviewing the manuscript. The authors also express their sincere gratitude to all the mothers who participated in this study.

**Conflict of interest:** Authors declare no competing interests.
